# Understanding the gap between guidelines and influenza vaccination coverage in people with diabetes: a scoping review

**DOI:** 10.3389/fpubh.2024.1360556

**Published:** 2024-04-19

**Authors:** Brice Mastrovito, Alexia Lardon, Amelie Dubromel, Viviane Nave, Karen Beny, Claude Dussart

**Affiliations:** ^1^Hospices Civils de Lyon, Pharmacie et Stérilisation Centrales, Pharmacie centrale, Saint-Genis-Laval, France; ^2^EA 4129 P2S Parcours Santé Systémique, Claude Bernard University Lyon 1, Lyon, France

**Keywords:** diabetes, influenza, vaccination, coverage, determinants, motivators and barriers, public health, pharmacist

## Abstract

**Background:**

Diabetes affects millions of people worldwide, making them more vulnerable to infections, including seasonal influenza. It is therefore particularly important for those suffering from diabetes to be vaccinated against influenza each year. However, influenza vaccination coverage remains low in this population. This review primarily aims to identify the determinants of influenza vaccination in people with diabetes (T1D or T2D). Secondly, it aims to assess main recommendations for influenza vaccination, vaccine effectiveness, vaccination coverage, and how education and pharmacists can encourage uptake of the vaccine in the diabetic population.

**Methods:**

A scoping review was conducted in January 2022 to systematically review evidence on influenza vaccination in people with diabetes using data from PubMed, Science Direct, and EM Premium with terms such as “Diabetes mellitus,” “Immunization Programs,” “Vaccination,” and “Influenza Vaccines.” Quality assessment and data extraction were independently conducted by two authors. Disagreements between the authors were resolved through discussion and consensus, and if necessary, by consulting a third author.

**Results:**

Of the 333 records identified, 55 studies met the eligibility criteria for inclusion in this review. Influenza vaccination was recommended for people ≥6 months. Despite effectiveness evidence showing a reduction in mortality and hospitalizations in people with diabetes vaccinated vs. non-vaccinated ones, very few studies reported a coverage rate ≥ 75%, which is WHO’s target objective. Determinants such as advanced age, presence of comorbidities and healthcare givers’ advice were associated with increased vaccination uptake. On the contrary, fear of adverse reactions and concerns about vaccine effectiveness were significant barriers. Finally, education and pharmacists’ intervention played a key role in promoting vaccination and increasing vaccination uptake.

**Conclusion:**

Influenza vaccination coverage in people with diabetes remains low despite recommendations and evidence on vaccine effectiveness. Motivators and barriers as well as several socio-demographic and clinical factors have been identified to explain this trend. Efforts are now needed to increase the number of diabetics vaccinated against influenza, mainly through education and the involvement of healthcare givers.

## 1 Introduction

Diabetes mellitus represents a significant global health challenge, affecting over half a billion people worldwide with its prevalence on an upward trajectory (1). The International Diabetes Federation (IDF) has documented an important increase in the global diabetes population: from 285 million in 2009 to 537 million in 2021, marking an approximate 90% surge in just over a decade (1–4). Projections suggest this number could reach 782 million by 2045 (1). As prevalence rises, so does the burden of disease, as evidenced by the number of diabetes-related deaths, estimated at 6.7 million in 2021 (1). Given its substantial contribution to healthcare costs, effective management of diabetes is essential. The global economic burden of diabetes, regardless of type, is estimated at more than 1.3 trillion dollars per year worldwide, primarily due to the management of diabetes-associated complications and hospitalizations (5). Among these complications, infectious diseases pose a significant risk, with people with diabetes experiencing higher susceptibility and severity of infections, including seasonal influenza (6, 7).

Prospective observational studies have consistently demonstrated the effectiveness of the influenza vaccine in reducing influenza-related complications and mortality in the diabetic population (8). Consequently, annual influenza vaccination is universally recommended for people with diabetes by health authorities (9, 10). Despite these recommendations, influenza vaccination coverage among the diabetic population remains low in many regions, significantly below the target objective of 75% set by European health authorities and the World Health Organization (WHO) (11, 12). Achieving this coverage could result in substantial healthcare savings, estimated between 190 and 260 million euros, through the reduction of influenza-related hospitalizations (13).

The discrepancy between vaccination guidelines and actual coverage rates, especially in the context of the ongoing COVID-19 pandemic and the risk of concurrent epidemics, underscores the critical need to understand and address the barriers to vaccination in the diabetic population. To date, literature reviews have synthesized the coverage and determinants of influenza vaccination in the general population, but none to our knowledge, has addressed its issues in people with diabetes. To fill this gap, this review primarily aims to identify the determinants of influenza vaccination in people with type 1 diabetes (T1D) or type 2 diabetes (T2D). Secondly, it aims to assess main recommendations for influenza vaccination, vaccine effectiveness, vaccination coverage, and how education and pharmacists can encourage uptake of the vaccine in the diabetic population. A better understanding of the current concepts and challenges may facilitate the development of improved strategies to increase influenza vaccination coverage in people with diabetes.

## 2 Methods

### 2.1 Literature search strategy

A scoping review approach was developed and used to systematically review evidence on influenza vaccination in people with diabetes (14). In a pilot search, multiple iterations of terms were queried to identify the most appropriate search terms, thus refining the search strategy and ensuring the selection of the most relevant literature. The literature search was conducted across PubMed, Science Direct, and EM premium using terms such as “Diabetes mellitus,” “Immunization Programs,” “Vaccination,” and “Influenza Vaccines.” The search results were restricted to articles in French or English, reflecting the language competencies of the readers. The search focused on articles published between January 1, 2002, and December 31, 2019. This period was strategically chosen to begin with the year following the American Diabetes Association’s 2002 report on influenza vaccination for people with diabetes, aligning with the WHO’s 2003 directive for achieving 75% influenza vaccination coverage by 2010 in populations with chronic diseases (15, 16). The endpoint of December 31, 2019, was selected to delineate the pre-COVID-19 era, acknowledging the pandemic’s significant disruption to influenza vaccination efforts and the comparability with subsequent data (17).

### 2.2 Selection of studies

Two review authors independently screened the titles and abstracts using a designed evaluation grid tailored to the review’s specific criteria and objectives. This structured approach facilitated the identification of studies potentially eligible for inclusion. After this initial screening, they independently assessed the full text of the chosen studies in detail, continuing to use the evaluation grid to ensure a systematic and thorough evaluation. Disagreements between the authors were resolved through discussion and consensus, and if necessary, by consulting a third review author. The results of the search, the inclusion of studies, and excluded studies and the reasons for their exclusion were reported in a PRISMA flow diagram (18).

### 2.3 Inclusion and exclusion criteria

Inclusion criteria included: full-text availability, study involving adult diabetic populations (T1D or T2D), cross-sectional, cohort, case–control studies, randomized controlled trial, quantitative or qualitative evaluation. Exclusion criteria included: non original research (i.e., letters or commentaries), meeting abstract, articles not focusing on influenza vaccination in diabetic populations, and articles not yielding relevant information for this review.

### 2.4 Study quality assessment

Scoping reviews are different from systematic reviews, as they include broader topics as well as studies with more diverse designs. Consequently, scoping reviews typically do not focus on the quality assessment of the included studies (19). Accordingly, the quality assessment of the included studies was not performed. However, this review includes the identification and explanation of the different biases encountered in the studies included.

## 3 Results

### 3.1 Identified literature

**Figure 1:**
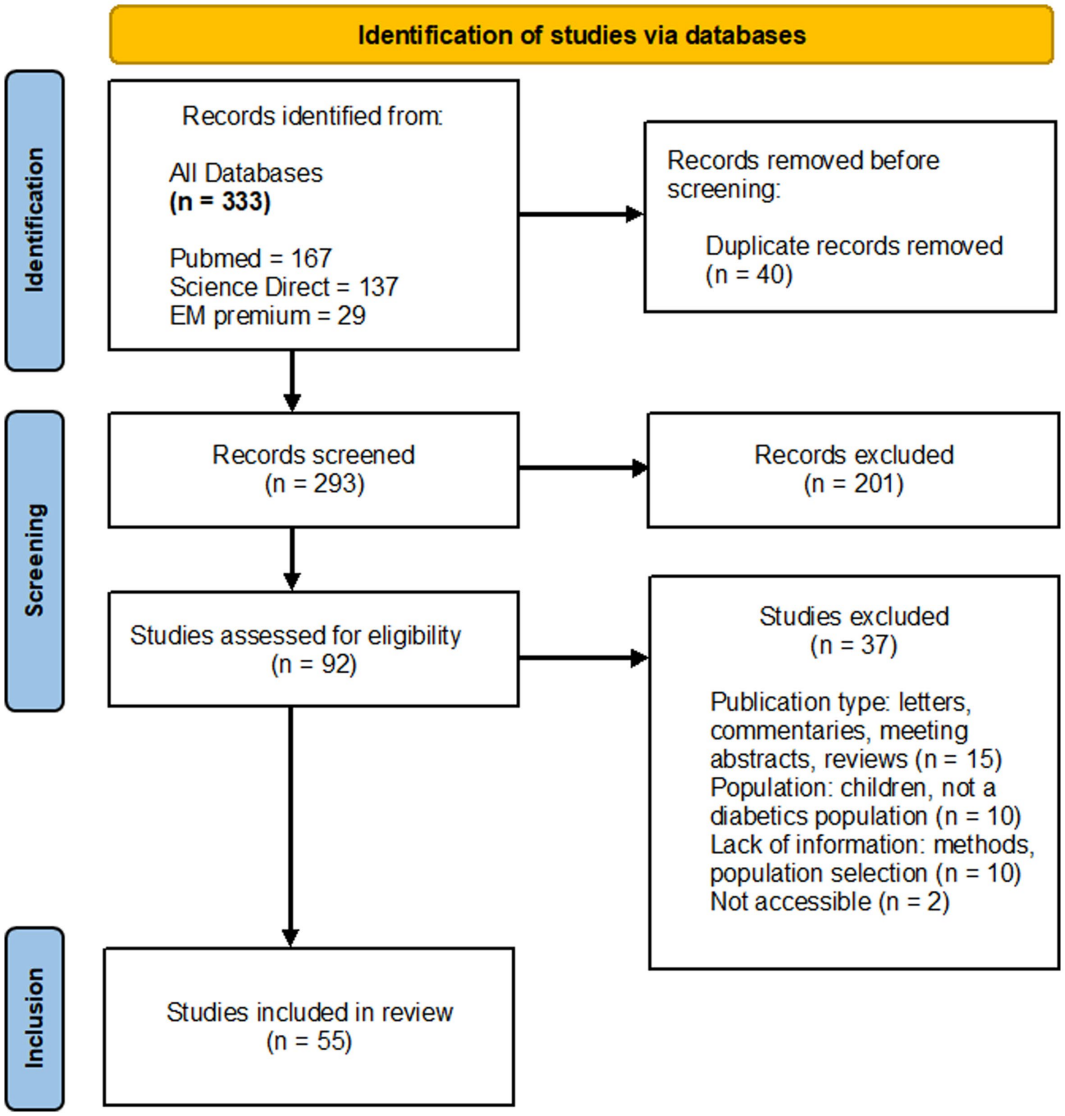
Flowchart of the study selection process.

The literature search led to the identification of 333 documents in the relevant databases described above (Figure 1). After duplicates were removed (*n* = 40), all remaining articles were first scanned by title and abstract. The full texts of 92 reports were retrieved, of which 55 met the inclusion criteria for the review. Of those reports retrieved but not included (*n* = 37), 15 studies were letters, commentaries, or meeting abstracts, 10 studies focused on children with diabetes or non-diabetic people, 10 studies lacked information on methods or population selection, two were not accessible.

### 3.2 Descriptive analysis of articles

**Figure 2:**
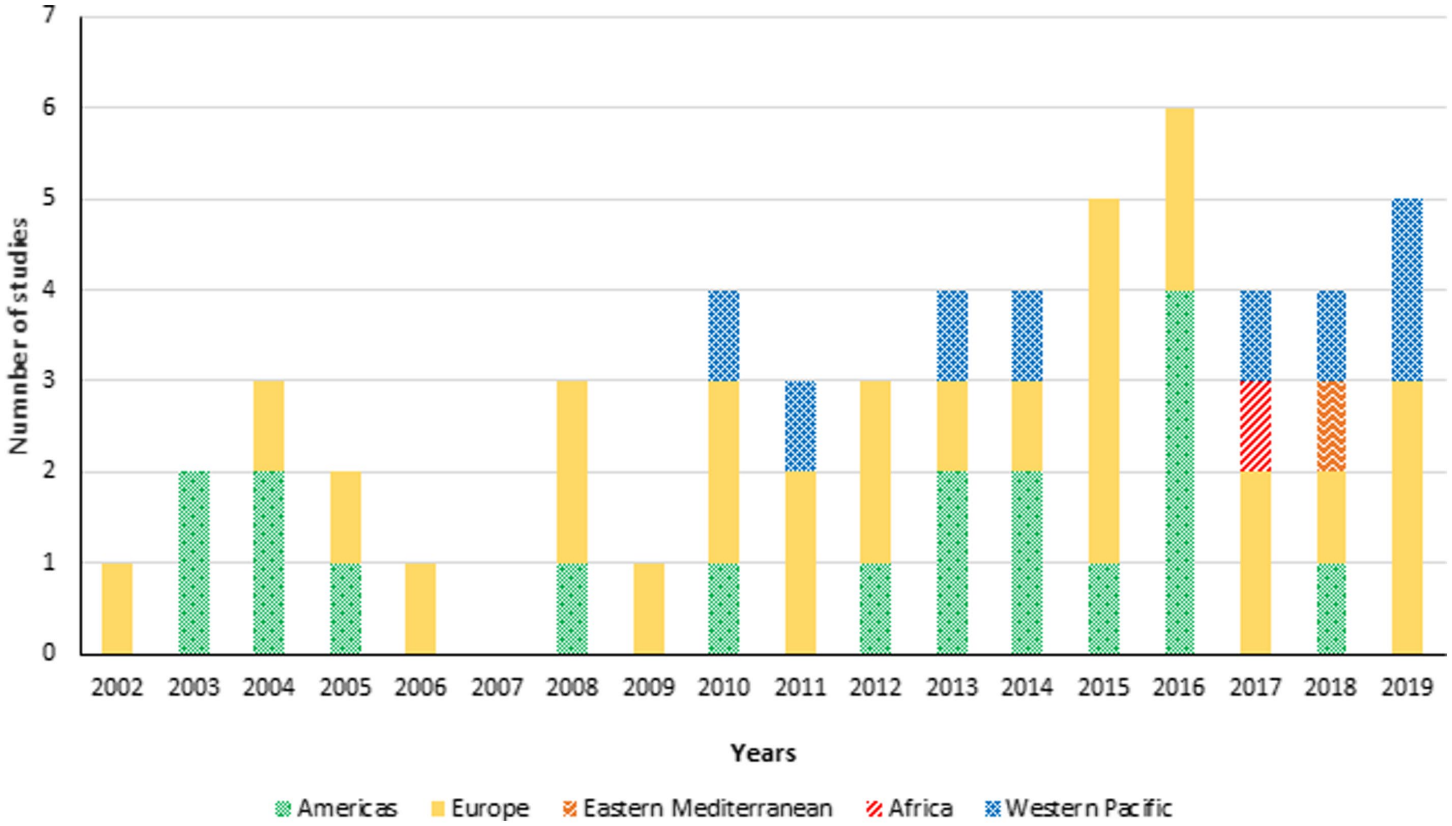
Total number of studies about influenza vaccination in persons with diabetes by year of publication (Jan. 1, 2002–Dec. 31, 2019) and region (*n* = 55).

Between January 1, 2002, and December 31, 2019, 55 studies of influenza vaccination in people with diabetes, from different WHO regions were included (Figure 2). The studies were unevenly distributed across the years and regions. The year with the highest number of studies was 2016 (*n* = 6), reflecting a growing interest in the topic. The early years (2002 to 2009) witnessed fewer studies, but there was a noticeable increase in the number of studies conducted each year after that. Most studies originated from the Americas and Europe, indicating a strong research interest in these regions. Other WHO regions (Africa, Eastern Mediterranean, Western Pacific) also contributed studies, though to a lesser extent.

Among the 55 studies included, four studied focused on current recommendations for influenza vaccination in diabetic populations, seven studies covered influenza vaccine effectiveness in people with diabetes, 38 studies assessed the influenza vaccination coverage rate and/or its determinants in diabetic populations, and six publications explored the role of education and pharmacist in promoting influenza immunization in people with diabetes.

### 3.3 Current recommendations for influenza vaccination

Four publications addressing vaccine recommendations in people with chronic diseases, and more specifically diabetes, were included in this review (20–23). The American Diabetes Association, in two separate articles published in 2003 and 2004, focused on the critical role of immunization in preventing influenza and pneumococcal disease in people with diabetes (20, 21). Similarly, in 2013, the Canadian Diabetes Association Clinical Practice Guidelines Expert Committee highlighted the importance of influenza and pneumococcal immunizations for people with diabetes (22). Building on this, a 2014 publication by the American Association of Diabetes Educators emphasized the essential need for hepatitis B, influenza, and pneumococcal vaccinations in people with diabetes (23). All these publications supported immunization for people with diabetes aged ≥6 months (20–23).

### 3.4 Influenza vaccine effectiveness

Seven publications on the effectiveness of influenza vaccination in people with diabetes were included in the review (Supplementary Table S1) (24–30). Three of these studies targeted individuals aged ≥65 years, while the remaining four also considered those <65 years. Heymann et al., observed a 12% decrease in All-Cause Hospitalization (ACH) rates for both T1D and T2D persons aged ≥65 years (27). A Canadian study involving participants aged ≥18 years reported the vaccine was associated to a 43% decrease in pneumonia and influenza hospitalizations, and a 28% decrease in ACH (24). A Dutch study, reported a 56% decrease in all complications, a 54% decrease in ACH, and a 58% decrease in All-Cause Mortality (ACM) for patients aged ≥18 years with T1D or T2D (26). Similarly, a Spanish study reported a 33% decrease in ACM among those aged ≥65 years (25). Further, Norwegian study reported a 78% decrease in influenza hospitalizations for individuals aged ≥30 years with T2D (28). In the United Kingdoms, vaccination was associated with lower hospitalization rates for stroke, heart failure, pneumonia, influenza, and ACM in patients aged ≥18 years with T2D (29). Lastly, Taiwanese study showed an 11% decrease in ACH and fewer intensive care admissions, alongside a decrease in ACM among those aged ≥65 years (30).

Three primary biases were identified in studies assessing the effectiveness of influenza vaccination in people with diabetes. Firstly, the “healthy vaccinee bias” indicated that vaccinated individuals typically engaged in healthier behaviors than their non-vaccinated counterparts. Such behaviors included more effective diabetes management, more frequent doctor visits, and greater attention to overall health. This bias could partly account for the observed lower mortality rates in vaccinated individuals, independent of the vaccination’s direct effects. To discern this bias, mortality rates outside the seasonal influenza epidemic periods were examined. Lower mortality rates in vaccinated versus non-vaccinated diabetic individuals during non-epidemic times suggested benefits not directly linked to influenza vaccination, as exposure to the virus was not a factor (24). Secondly, a bias concerning pneumococcal vaccination status was noted. Individuals regularly vaccinated against influenza were also more likely to receive pneumococcal vaccinations. Rodriguez et al. found that among diabetic individuals vaccinated annually for influenza, 70% had also received a pneumococcal vaccine, compared to only 22% among those not vaccinated for influenza (25). Given that pneumococcal pneumonia is a common complication of seasonal influenza, a reduction in hospitalizations and mortality rates might have been more accurately attributed to pneumococcal rather than influenza vaccination. Lastly, selection bias was observed, wherein diabetic individuals with comorbidities are more frequently vaccinated. These comorbidities were associated with an increased risk of complications, possibly leading to an underestimation of the influenza vaccine’s effectiveness (26). The lack of distinction between T1D and T2D also introduced bias. Individuals with T2D, who were more prone to complications from an influenza infection due to multiple comorbidities, may have skewed effectiveness results (25–27).

### 3.5 Prevalence of influenza vaccination

Despite recommendations advocating for influenza vaccination in people with diabetes, actual vaccination coverage remained relatively low across regions (20–23). Thirty-one studies focused on assessing influenza vaccination coverage in the diabetic population (Supplementary Table S2) (31–61). In Europe, vaccination coverage has shown variability: the Netherlands reported a high of 85% in 1999, with a decline to 75% by 2013 (44, 45). Spain’s coverage fluctuated between 34 and 66% from 1993 to 2018 (47–49, 51–53, 55, 56), while Belgium saw an increase from 46% in 2006 to 49% in 2010 (40–42). Ireland and Switzerland maintained rates around 65% (43, 57), contrasting with Poland’s lower 27% (46), and Austria’s range of 6% in 1991 to 21–22% in 2006–2007 (39). In the Americas, the United States experienced coverage rates between 49 and 62% (33–37), while Brazil showed an upward trend to 59% in 2015 (31), and Canada reported a 63% coverage rate (32). The Western Pacific region saw Australia and Taiwan with rates around 30–35% (58, 61), while China and Korea reported higher rates of approximately 50–55% (59, 60). In the Eastern Mediterranean, Saudi Arabia reported a 61% coverage rate (38).

The main bias for the aforementioned studies was the assessment of vaccination status via a questionnaire or survey for some of them. This approach introduced a social-desirability bias, where individuals might have claimed to be vaccinated to align with the positive perception of vaccination, especially since influenza vaccination is strongly recommended for people with diabetes. Such a tendency to report vaccination favorably, regardless of actual vaccination status, may have led to inflate the estimated vaccination rate in some of the studies (47, 52, 54). Another significant bias was nonresponse bias, which occurred when the responses collected represented only a portion of the target group, with some studies noted non-responder rates of 30–50% (52). This bias raised concerns about the accuracy of the results, as the true vaccination coverage might have been different if all individuals had responded. Memory bias also played a crucial role, as participants were asked to recall their vaccination history. This bias may have led to inaccuracies, particularly for vaccinations received in more distant years, affecting the reliability of reported vaccination rates (61). Additionally, the challenge of underdiagnosis of diabetes in some populations, coupled with the inadequate distinction between T1D and T2D, introduced further biases into the studies (47).

### 3.6 Determinants of influenza vaccination

#### 3.6.1 Barriers and motivators

**Figure 3:**
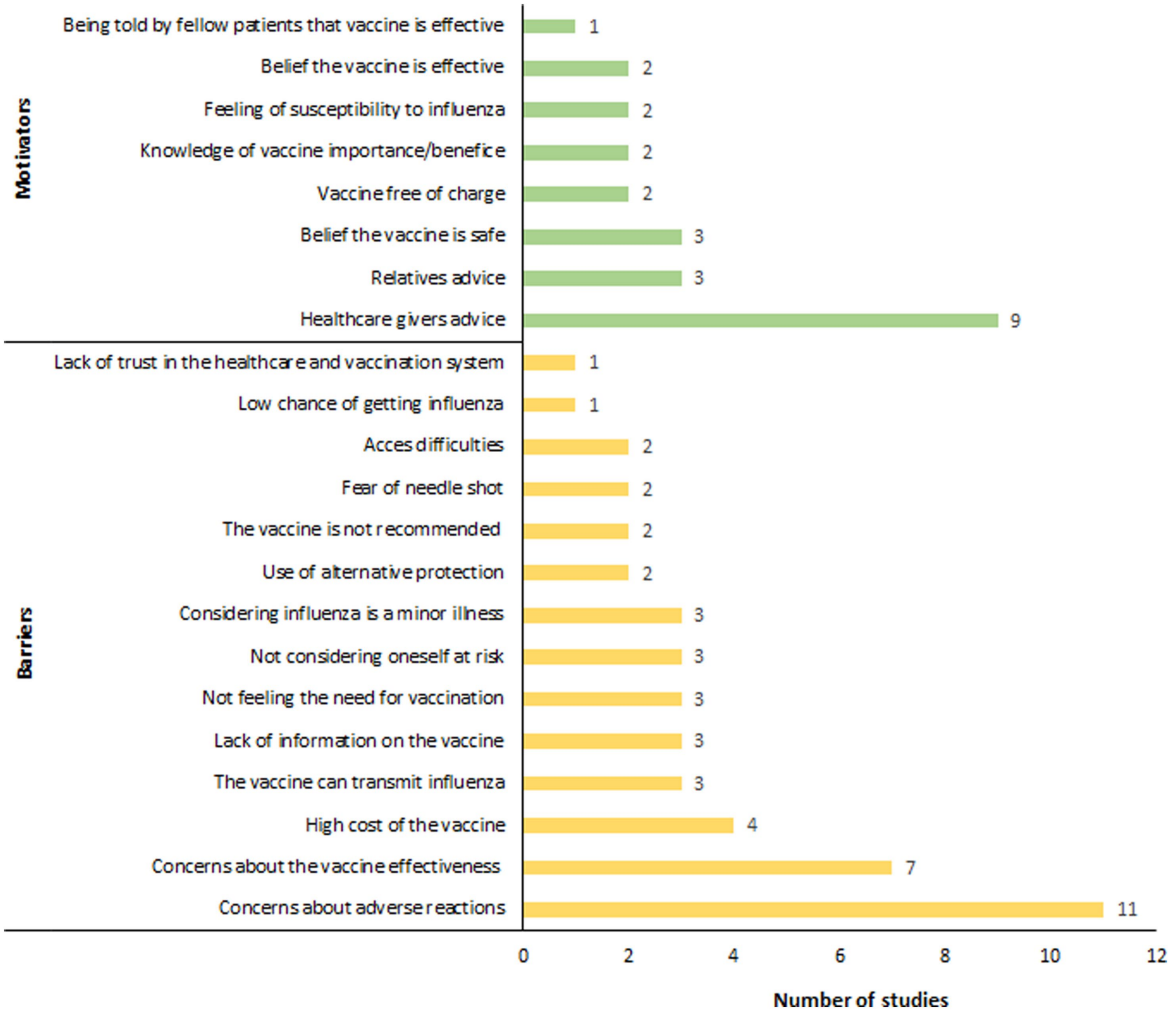
Barriers and motivators concerning influenza vaccination for people with diabetes.

Twenty-four studies investigating the determinants influencing influenza vaccination coverage in people with diabetes were included in this review (31, 32, 35, 37, 38, 43, 46, 47, 51, 52, 54–56, 58–68) (Figure 3). A primary barrier identified was concern over adverse reactions, which disproportionately affected women (54, 63). Jiménez-García et al. found that fear of adverse reactions was a significantly more common reason for vaccine avoidance among women (32.5%) compared to men (20.2%) (54). Risk perception-related reasons were also commonly cited as barriers to influenza vaccination. People with diabetes who reported no need for the vaccine (47, 65, 66), perceived themselves to be in good health or not at risk (46, 54, 62), and considered influenza as a minor illness (38, 54, 64), were less inclined to get vaccinated. In regions where influenza vaccination was not financially covered for people with diabetes, vaccine cost was also reported as a major barrier to vaccination (46, 62, 64, 66). Moreover, a lack of information about influenza vaccination (46, 62, 66), particularly concerning safety and effectiveness of the vaccine, along with misconceptions (e.g., the belief that the vaccine could cause influenza) (38, 54, 65), were also major obstacles to vaccination. Finally, the controversy surrounding the 2009 H1N1 influenza pandemic further eroded trust in the pharmaceutical industry and health authorities, leading to decreased vaccination rates in the diabetic population in some regions, notably France, since 2009 (63).

On the other hand, factors emerged as significant motivators for influenza vaccination in people with diabetes (Figure 3), with advice from healthcare providers standing out as a critical influence. A substantial proportion of vaccinated individuals, ranging from 80 to 90%, reported following their physician’s recommendation as the reason for getting vaccinated (38, 46, 47, 54, 59, 62, 64–66). Besides healthcare advice, recommendations from relatives (38, 62, 66), exposure to media campaigns (38), and personal narratives from already vaccinated individuals also played notable roles in motivating people to seek vaccination (64). A deep understanding of the importance of vaccination was also identified as a key motivator (38, 61). Olatunbosun et al. reported that individuals with a comprehensive understanding of both seasonal influenza and its vaccination were 3.8 times more likely to be vaccinated compared to those with limited knowledge (64). Additional motivators included the perceived safety (59, 61, 62), and effectiveness of the vaccine (59, 62), vaccine free of charge (62, 64), and a personal feeling of susceptibility to influenza (52, 62).

#### 3.6.2 Socio-demographics factors

**Figure 4:**
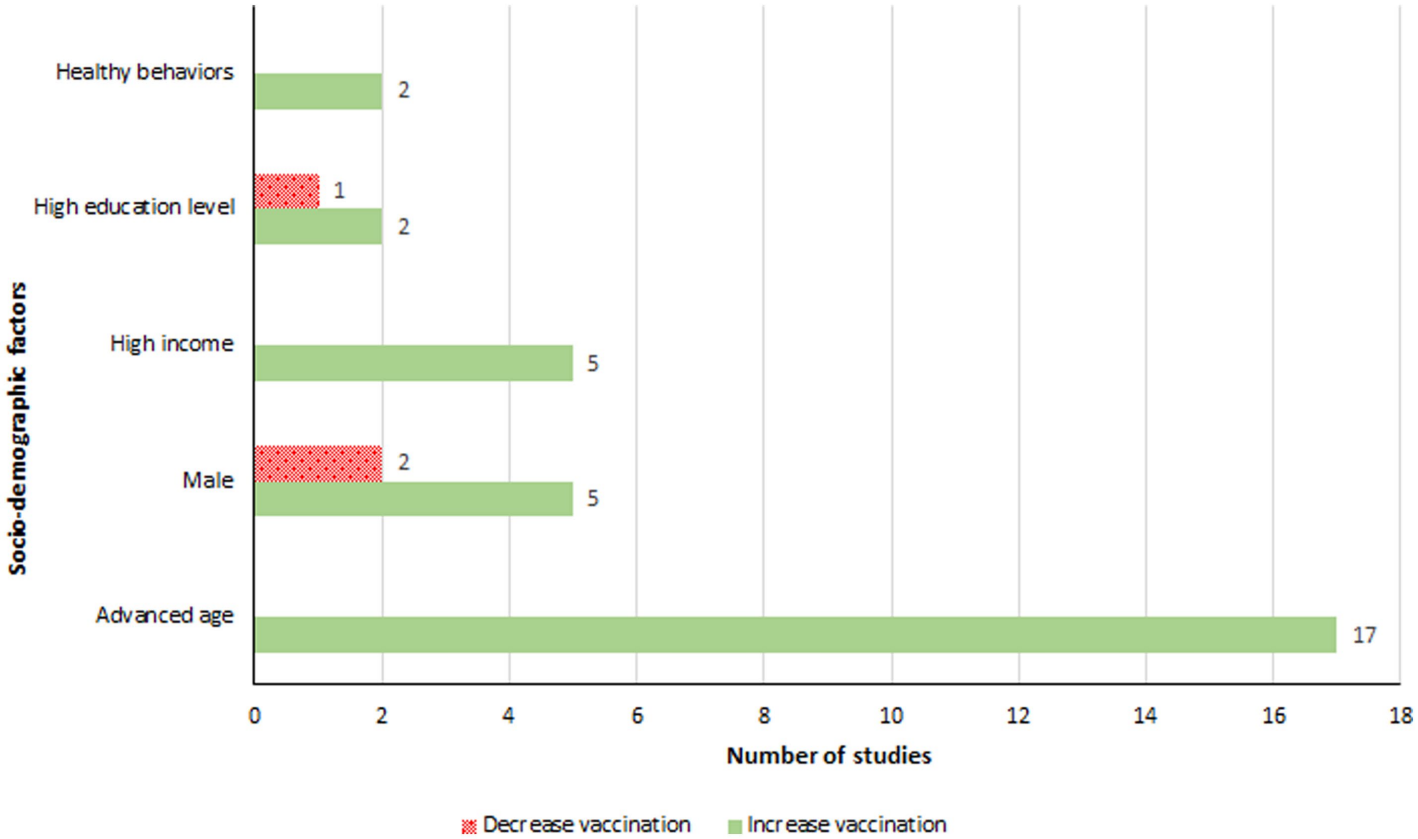
Socio-demographic factors associated with an increase or a decrease in influenza vaccination.

Age was a significant factor for influenza vaccination in people with diabetes, with studies reported higher age was associated with increased vaccination rates (31, 32, 37, 43, 47, 51, 52, 54–56, 58, 60, 61, 63, 64, 67) (Figure 4). Shin et al. found that 79% of individuals aged ≥65 years were vaccinated, compared to 34% in the 40–64 age group (60). Jimenez-Trujillo et al. observed that the likelihood of vaccination in diabetic individuals aged 60–69 was more than triple that of those aged 50–59, and around six times greater for those aged ≥70 years (52). Gender also influenced vaccination coverage, with male diabetic patients in Spain showing increased vaccination rates (47, 48, 52). However, studies from Korea and Taiwan reported a decrease in vaccination rates among males (60, 61). Additionally, higher income groups were associated with increased vaccination coverage (37, 46, 60, 66, 67). Tan et al. highlighted that individuals earning more than $4,000 per month were three times more likely to get vaccinated than those earning below $2,000 (66).

#### 3.6.3 Clinical factors

**Figure 5:**
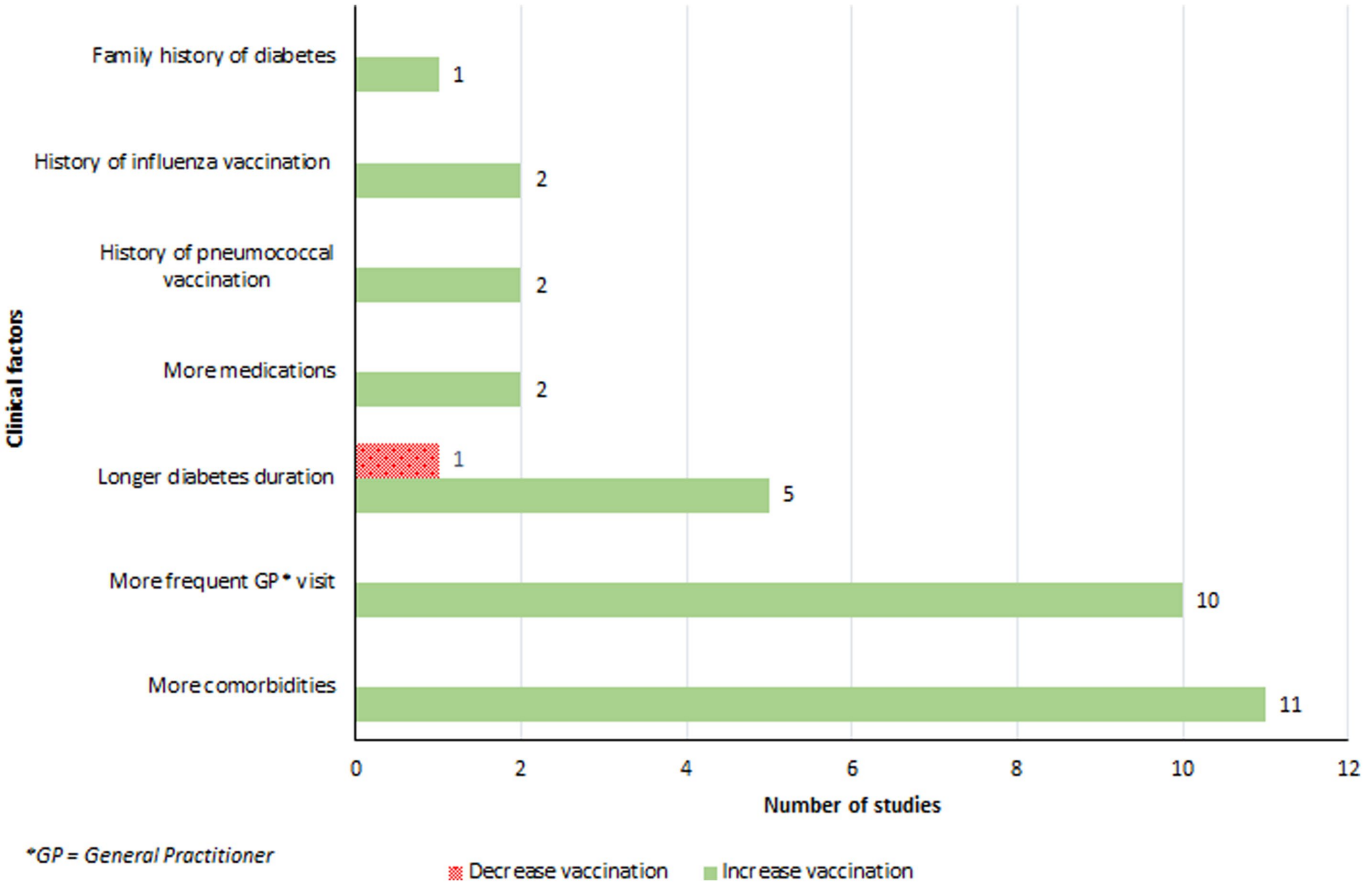
Clinical factors associated with an increase or a decrease in influenza vaccination.

The presence of comorbidities, particularly lung and cardiac diseases, significantly influenced vaccination coverage (32, 38, 46, 47, 51, 52, 54, 61, 67) (Figure 5). Patients who had recent or frequent consultations with their primary care physician were more likely to receive the influenza vaccine (32, 35, 43, 47, 51, 52, 54, 60, 67, 68). Vaccinated patients reported visiting their general practitioner on average 2.5 times more in the previous year compared to their non-vaccinated counterparts (54). An increase in vaccination coverage was also observed among individuals with a longer duration of diabetes (43, 54, 58, 64, 67). Olatunbosun et al. found that study participants with diabetes for 6–10 years were 4.3 times more likely to be vaccinated than those diagnosed for 5 years or less (64). There was a positive association between the number of medications prescribed to an individual and vaccination rates, with vaccinated individuals taking more medications for their diabetes or other conditions than those who were not vaccinated (32, 46). Moreover, a history of influenza (54, 62), or pneumococcal vaccine (35, 54), significantly increased the likelihood of vaccination. Lastly, a family history of diabetes was linked to higher vaccination rates (38).

#### 3.6.4 Biases encountered

Several biases have been identified in studies examining the determinants of influenza vaccination among individuals with diabetes, with some overlaps seen in studies on vaccination coverage. Primarily, utilizing questionnaires for data collection introduced memory bias, non-response bias, and social-desirability bias (51, 52, 54, 60, 64, 67). Additionally, a notable issue in some studies was the lack of distinction between T1D and T2D (51, 60, 65). This oversight was significant given the distinct characteristics of T1D and T2D populations, especially concerning age. Consequently, the potential differences in vaccination barriers and motivators between younger and older individuals were not adequately explored, leading to biased interpretations of the results. Moreover, the study populations often did not represent the general diabetic population well, limiting the generalizability of the findings to all individuals with diabetes (60, 62, 64, 66). Finally, it is crucial to recognize that the decision to get vaccinated is dynamic, subject to change over time within an individual (54). Conducting studies on this topic is complex, as vaccination adherence is shaped by an individual’s perceptions, as well as by psychosocial and motivational factors.

#### 3.6.5 Empowering influenza vaccination through education: the key role of pharmacists

Six studies highlighted the importance of education in increasing vaccination coverage among people with diabetes (69–74). Firstly, one notable study by Altay et al. demonstrated that patient education significantly boosted influenza vaccination rates across various age groups, increasing from 12.1 to 36.6% (73). Another significant study by Tao et al. involved a comprehensive community-based approach, encompassing 1,538 diabetic patients. This study used general practitioners to conduct educational interventions, resulting in an increase in vaccination rates within the intervention group, from 29.0 to 45.8% (74). Secondly, four studies assessing the role of pharmacists in promoting influenza vaccination among individuals with diabetes were included. First study showed that 41.7% of patients without a prior record of influenza vaccination received the vaccine within a 12-month evaluation period, facilitated through personalized diabetes education and medication consultations by pharmacists (71). Similarly, Fera et al. documented an increase in vaccination rates from 43 to 61% following the engagement of community pharmacists, who had undergone a diabetes certification program and provided scheduled consultations and health monitoring (70). This trend was echoed in another study, which witnessed a significant rise in vaccination rates from 52 to 77%, attributed to comparable interventions by community pharmacists (72). Additionally, Miller et al. adopted a novel strategy by utilizing pharmacy students to deliver educational interventions to patients, significantly increasing the percentage of patients comfortable with receiving the H1N1 influenza vaccine from a pharmacist from 69.3 to 81.4% (69).

## 4 Discussion

Effective management and reduction of influenza infections in people with diabetes depends on the uptake of the influenza vaccine. Significant advancements have been achieved in the development of vaccines that are safe and effective. However, having these vaccines is not enough. Implementing vaccination programs for these people involves overcoming challenges such as logistics concerns, cost, and equitable distribution, alongside promoting vaccine acceptance within the diabetes community. An increasing hesitancy towards vaccination in people with diabetes pose significant obstacles to global efforts aimed at mitigating the spread and impact of influenza among this at-risk group.

This review aims to highlight the inadequacy of influenza vaccination in people with diabetes, despite international recommendations. It provides insights into vaccination coverage worldwide and explored factors affecting uptake in people with diabetes, thus enabling further measures to be identified to improve vaccination coverage in this population. Nevertheless, this review has several limitations. Firstly, selection criteria were limited to studies published in English or French, due to the language proficiency of the readers. This language restriction led to the exclusion of a total of 24 of the original 357 studies identified by the searching equation. Among these, only minority could have been considered eligible for inclusion in this review. Thus, although including all identified studies regardless of language would have been ideal for a comprehensive analysis, the final impact on the results of this review is likely to be limited given the small proportion of potentially relevant studies that were excluded. Secondly, the origin countries of the included studies were categorized according to the six regions defined by the WHO to provide a framework consistent with global health divisions that may influence regional public health strategies, as each region has a WHO office focusing on disease prevention and control strategies. However, it is important to acknowledge that within these WHO regions, countries may face unique challenges regarding influenza vaccination influenced by economic, cultural, and systemic factors. Thirdly, this review may be subject to publication bias. While countries like Spain and the United States are well-represented, others with significant contributions to vaccine policies, such as Portugal are missing. Notably, Portugal was one of the first countries to introduce vaccination by pharmacists in 2007 (75, 76). Fourthly, the included studies addressing vaccination recommendations were all North American, leading to an underrepresentation of other regions of the world and thus a poor view of international recommendations. Fifthly, while figures highlighted the number of studies addressing the different determinants of vaccination, a presentation involving the number of people associated with each determinant would have seemed more appropriate to have a more comprehensive picture. However, the representation of the number of subjects could have suggested that a quantitative analysis was carried out, which is not the case, and would have not been consistent with the aim of a scoping review. Finally, the potential methodological heterogeneity of the included studies, due to the lack of a systematic quality assessment, could affect the interpretation and reliability of the findings.

This review found that the recommendation for influenza vaccination applied to people with T1D or T2D, starting from the age of 6 months. However, only North American studies were identified, suggesting a need for more geographically diverse research (20–23). Across the Atlantic, the European Centre for Disease Prevention and Control (ECDC) also recommends yearly influenza vaccination for people with chronic medical conditions, including diabetes, as part of a broader strategy to safeguard those most susceptible to severe influenza (77). The National Health Service (NHS) in the UK holds a similar position, advising that adults with conditions such as diabetes receive the influenza vaccine each year (78). Likewise, France recommends influenza vaccination annually for people with diabetes (79, 80), while in Australia, the government provides the influenza vaccine free of charge to high-risk groups including people with diabetes (81). These recommendations from major health organizations globally underscore the crucial role of influenza vaccination in protecting people with diabetes. However, despite these guidelines, there is still considerable variation in vaccination rates in people with diabetes, indicating a pressing need for intensified public health initiatives to enhance vaccine uptake.

Results presented in this review highlighted several benefits of influenza vaccination in people with diabetes. They suggested that the vaccine significantly decreased rates of all-cause mortality and hospitalizations, including specific reductions in influenza and pneumonia hospitalizations (24–30). These findings align with earlier evidence of the influenza vaccine’s effectiveness in diabetic populations, notably a study by Colquhoun et al. that reported a 79% reduction in hospital admissions for vaccinated diabetic patients compared to their non-vaccinated peers across two influenza seasons (82). However, several biases common to most studies on the effectiveness of influenza vaccination in people with diabetes were identified, the main one being the healthy vaccinee bias (24). This bias is also reported and described in other studies and can lead to an overestimation of the benefits attributed to influenza vaccination (83–85). Another significant bias identified was related to the pneumococcal vaccination status. It was noted that a majority of individuals vaccinated against influenza had also received the pneumococcal vaccine (25). Consequently, some of the benefits attributed to influenza vaccination may have been due to the pneumococcal vaccine (86, 87). Moreover, there was frequently no differentiation made between T1D and T2D in studies included. This oversight introduced an additional layer of bias, as individuals with T2D, who often have several comorbidities, are more susceptible to complications after an influenza infection (25–27).

Despite existing recommendations advocating for influenza vaccination in people with diabetes, coverage rates remained low in numerous regions. The heterogeneity observed in reported results can be attributed to various factors, including differing study inclusion criteria, demographic considerations, and the specific years in which studies were conducted. Few studies reported vaccination coverage rates meeting or exceeding the 75% target set by the WHO for this population (16). In Europe, the Netherlands achieved an 85% coverage rate in 1999 (44), yet witnessed a decline to 75% by 2013 (45). Spain (47–56), and Belgium (40–42), showed considerable fluctuations in their vaccination rates, whereas Poland (46), and Austria (39), reported notably lower figures. In the Americas, coverage in the United States ranged between 49 and 62% (33–37), while Brazil displayed a positive trend, reaching 59% in 2015 (31). In the Western Pacific, Australia and Taiwan had coverage rates of approximately 30–35% (58, 61), in contrast to China and Korea, where rates hovered around 50–55% (59, 60). The Eastern Mediterranean region saw Saudi Arabia reporting a 61% coverage rate (38). In estimating the influenza vaccination coverage rate, several biases were reported, largely stemming from questionnaire-based data collection methods. Social desirability bias may have inflated reported vaccination rates, as some individuals could affirm vaccination status to be viewed more favorably (47, 52, 54). Non-response bias occurred when the respondents of a survey did not represent the entire target population, potentially skewing results (52). Memory bias, particularly concerning recollections of past vaccination statuses, could compromise accuracy, especially for inquiries regarding distant past events (61). Furthermore, the under-diagnosis of diabetes in certain locales, coupled with inadequate differentiation between T1D and T2D, introduced additional layers of complexity to accurate vaccination coverage assessment (47).

A range of factors that influence influenza vaccination rates among individuals with diabetes has been identified. These factors were categorized into barriers, motivators, socio-demographic elements, and clinical aspects, highlighting the complex nature of vaccine acceptance and adherence. One of the primary barriers identified was concern over adverse reactions (38, 43, 46, 54, 55, 59, 61–65), indicating significant apprehension. Additionally, self-perceived risk of contracting influenza played a crucial role in vaccination decisions. Diabetic individuals who rated their health status highly were less likely to get vaccinated compared to those with a lower health status assessment (38, 46, 47, 52, 54, 62, 64–66), aligning with literature that underscores the impact of self-perceived risk on vaccination rates (88). This emphasizes the need for adequate health education about the risks of influenza, especially for diabetes patients. Economic considerations, like the cost of the vaccine, also emerged as significant factors, particularly in regions where vaccination were not subsidized (46, 62, 64, 66). Furthermore, knowledge about influenza and its vaccine was critical. The spread of misinformation, lack of information, and misconceptions posed major barriers to vaccination (46, 62, 66). This situation underlined the importance of effective, transparent, and accessible communication strategies to address these barriers. Additionally, this review highlighted the importance of trust in the pharmaceutical industry and government health organizations. Vaccination rates declined following the 2009 H1N1 influenza pandemic, a phenomenon partly due to healthcare controversies in France that eroded public trust in health agencies, including skepticism towards the influenza vaccine. Since the pandemic, a noticeable portion of the French diabetic population has been reluctant to get vaccinated (63). One study highlighted the significant negative impact of the 2009 H1N1 pandemic on French public attitudes towards vaccines, with 41% expressing a poor opinion of them (89). This skepticism is not unique to France but is evident across Europe, although recent studies indicated that vaccine confidence has been on the rise since 2015 (90). Physicians played a critical role in influencing influenza vaccination rates, with patients showing high receptiveness to their advice (38, 46, 47, 54, 59, 62, 64–66). By sharing their expertise and insights on influenza vaccination, physicians enable patients to make informed decisions based on scientific evidence and the latest health guidelines. However, not all physicians view the influenza vaccine as effective (91, 92), with one study revealing that 50% of physicians had not received the influenza vaccine in the decade prior to the survey (93). Importantly, physicians who are vaccinated against influenza are more inclined to recommend the vaccine to their patients (94, 95), although 10% believe their endorsement is not crucial (93). It is imperative that physicians, especially those in primary care, consistently recommend influenza vaccination to their diabetic patients. Further research is needed to understand the factors that motivate or deter physicians from recommending the influenza vaccine. Insights from such studies could identify ways to encourage physicians to recommend the vaccine, potentially increasing coverage among diabetic patients (92, 93). Regular visits to primary care providers were associated with higher influenza vaccination rates among diabetics (47, 51, 52, 54, 60, 63, 67), likely because frequent consultations provide more opportunities for vaccination discussions. Looking ahead, it is crucial to emphasize the importance of influenza vaccination. Evidence shows that vaccination rates improve through various methods, such as displaying posters in waiting areas or through discussions with healthcare professionals (physicians, nurses, and pharmacists) (96–98). Additionally, sending vaccination reminders to both patients and their physicians has been proven to effectively increase vaccination rates (96, 99, 100).

Socio-demographic factors significantly influenced influenza vaccination decisions. Notably, advanced age was associated with higher vaccination rates, likely due to an increased awareness of the risks associated with influenza in older age groups (31, 32, 37, 43, 47, 51, 52, 54–56, 58, 60, 61, 63, 64, 67). Individuals with diabetes often received vaccinations against influenza due to their age rather than their diabetic condition itself (61). Indeed, several studies have indicated that diabetic individuals did not perceive influenza as having more severe implications for them compared to non-diabetic individuals (61, 64). Given these findings, reconsidering the age threshold for routine influenza vaccination could be beneficial (101). The impact of gender on vaccination rates has yielded mixed results. The review found that in Spain, fewer women were vaccinated compared to men (47, 48, 52), whereas in Korea and Taiwan, the opposite trend was observed, with more women receiving the vaccine (60, 61). One explanation might be that men generally have more comorbidities, making them priority candidates for influenza vaccination (67). This is supported by evidence suggesting that physicians are more likely to recommend influenza vaccinations to patients with comorbidities (92), a factor also associated with higher vaccination rates among individuals with diabetes in this review (32, 38, 46, 47, 51, 52, 54, 61, 67). Further investigation is needed to understand these gender disparities fully, but it is crucial that influenza vaccination promotion does not discriminate by gender. Additionally, this review highlighted an association between higher income levels and increased vaccination rates, pointing to economic barriers as a significant obstacle for lower-income groups (37, 46, 60, 66, 67). This emphasizes the need for strategies to make influenza vaccinations more accessible to individuals across all income brackets.

Clinical factors played a pivotal role in the decision-making process for influenza vaccinations. Patients with comorbidities, especially those suffering from cardiac and pulmonary diseases (32, 38, 46, 47, 51, 52, 54, 61, 67), as well as those who frequently visit their primary care physicians, showed a higher propensity towards vaccination (32, 35, 43, 47, 51, 52, 54, 60, 67, 68). Additionally, a longer duration of diabetes was associated with an increased likelihood of getting vaccinated, possibly due to more frequent interactions with the healthcare system or a heightened awareness of health risks over time (43, 54, 58, 64, 67).

It is crucial to acknowledge that biases such as memory, non-response, and social desirability, along with challenges in differentiating between T1D and T2D, may have impacted the outcomes of the analyzed studies. The dynamic nature of vaccination decisions, which can evolve over time, adds another layer of complexity to enhancing vaccination rates.

This review highlighted the critical role of education in boosting influenza vaccination coverage among individuals with diabetes. This aligns with prior research emphasizing education as a key element in fostering adherence to preventive healthcare practices (102, 103). Studies by Altay et al. and Tao et al. demonstrated the positive effect of patient education on vaccination rates, supporting the notion that increased knowledge and understanding of vaccination benefits and safety can markedly improve vaccine acceptance and uptake (73, 74). Furthermore, the involvement of pharmacists, as evidenced by four studies, underscored the value of a multidisciplinary healthcare team approach to elevate vaccination rates. The integration of pharmacists into diabetes care teams, offering personalized education and medication consultations, highlighted their vital role in enhancing patient education. This has not only boosted patient comfort and confidence in vaccinations, but has also underscored the importance of pharmacists’ active participation in vaccine advocacy (69, 70). In many countries, pharmacists have been granted the authority to administer influenza vaccinations, a move aimed at supporting physicians and enhancing vaccination rates (104, 105). Over recent years, pharmacists have significantly transformed their role, emerging as key figures within the public health domain (106). The decision to allow pharmacy-based influenza vaccinations has correlated with increased vaccination uptake, a trend that has strengthened over time (107–112). This rise in vaccination rates can be attributed to several advantages associated with community pharmacies. Firstly, the convenience of community pharmacies has been widely acknowledged (113–115). As the most accessible healthcare professionals, pharmacists often serve as the first point of contact for healthcare inquiries. Community pharmacies are well-distributed, covering both urban and rural areas where medical services may be less accessible, and they tend to have flexible operating hours (116, 117). This accessibility is particularly beneficial for increasing vaccination rates among individuals who might not typically seek vaccination (117). Additionally, community pharmacies are well-positioned to target at-risk groups, such as individuals with diabetes, more effectively. Pharmacists can identify those eligible for influenza vaccinations through pharmacy records and patient consultations. Given that individuals in at-risk groups often visit community pharmacies to refill prescriptions, these visits provide convenient opportunities for vaccination (118). Papastergiou et al. found that 21% of high-risk individuals reported they would not have been vaccinated if the service were not available at community pharmacies (113). Moreover, satisfaction rates for pharmacy-based vaccinations exceed 95%, with patients endorsing the pharmacists’ new role (119, 120). The preference for community pharmacy vaccinations is not only due to convenience but also the perception of community pharmacies as less stressful environments with a lower risk of exposure to sick individuals (121). The COVID-19 pandemic has further underscored the value of community pharmacy-based vaccinations, as concerns over exposure in traditional healthcare settings have led to a reduction in visits to primary care providers (122). Patients are reassured by the expertise of pharmacists and their capability to safely administer vaccinations, which aligns with pharmacists’ roles in educating and advising on vaccination matters (121). With the authorization to administer influenza vaccines in many countries, pharmacists are well-placed to help achieve vaccination coverage objectives set by organizations like the WHO. The strategic positioning and recognized expertise of community pharmacists make them essential players in vaccinating at-risk populations, including those with diabetes. Given the international emphasis on vaccinating priority groups against influenza, leveraging the role of pharmacists in this effort is crucial.

## 5 Conclusion

This scoping review synthesized current insights into influenza vaccination among individuals with diabetes. To date few publications have focused on this topic. Despite international recommendations supporting influenza vaccination in people with diabetes and its proven effectiveness, coverage has varied significantly over time and by region but has remained low. Determinants such as being older, the presence of comorbidities, frequent visits to the physician, a history of vaccination as well as good knowledge of the influenza vaccine have been associated with greater vaccination uptake. Conversely, a good self-rated health status, poor perception of the risk of complications and fear of adverse events have all been associated with lower vaccination uptake. Healthcare professionals, especially pharmacists who are among the main initiators of patient education in healthcare, should increase their efforts to educate about and promote influenza vaccination in individuals with diabetes. Moving forward, a focused approach on these determinants and a global commitment to enhancing patient education may significantly improve vaccination rates. Such efforts are essential for better protecting people with diabetes against influenza.

## Data availability statement

The original contributions presented in the study are included in the article/Supplementary material, further inquiries can be directed to the corresponding author.

## Author contributions

BM: Conceptualization, Data curation, Formal analysis, Methodology, Software, Writing – original draft. AL: Data curation, Formal analysis, Methodology, Writing – review & editing. AD: Conceptualization, Methodology, Supervision, Validation, Writing – review & editing, Formal analysis. VN: Methodology, Supervision, Writing – review & editing. KB: Methodology, Supervision, Writing – review & editing. CD: Conceptualization, Methodology, Project administration, Supervision, Writing – review & editing, Validation, Formal analysis.
