# Supplementary material for: Understanding the gap between guidelines and influenza vaccination coverage in people with diabetes: a scoping review
Source: Front Public Health. 2024 Apr 19;12:1360556. doi: 10.3389/fpubh.2024.1360556 (PMC11066301; doi:10.3389/fpubh.2024.1360556)
Supplement: Supplementary file 1 [file Table_1.PDF]

Table S1. Summary table of studies included on the effectiveness of influenza vaccination in people with diabetes

Reference	Country	Study design	Influenza season	Diabetes type	Population age	Population	Subgroups	Main outcomes
Heymann et al, 2004	Israel	Outcome-research study	1999-2000	Type 1 & 2	≥65 years	n = 15,556	None	↘ 12% in ACH
Lau et al, 2012	Canada	Retrospective cohort study	2000-2008	Type 1 & 2	≥18 years	n = 56,513	18-64 years	↘ 43% in PIH (95% CI: 28%-54%)<br>↘ 28% in ACH (95% CI: 24%-32%)
							≥65 years	↘ 45%-55% in PIH<br>↘ 33%-34% in ACH
Looijmans-van den Akker et al, 2006	Netherlands	Nested case-control study	1999-2000	Type 1 & 2	≥18 years	n = 9,238	None	↘ 56% all complication (95% CI: 36–70%)<br>↘ 54% in ACH (95% CI: 26-71%)<br>↘ 58% ACM (95% CI: 13-80%).
Rodriguez-Blanco et al, 2012	Spain	Retrospective cohort study	2002-2005	Type 1 & 2	≥65 years	n = 2,650	None	↘ 33% in ACM (95% CI: 4%-53%)
Ruiz et al, 2020	Norway	Retrospective cohort study	2009-2013	Type 2	≥30 years	n = 2,992,228	None	↘ 78% in IH (aHR = 0.22, 95% CI: 0.11-0.39)<br>↘ 25% in ACM (aHR = 0.75, 95% CI: 0.73-0.77)
Vamos et al, 2016	UK	Retrospective cohort study	2003-2010	Type 2	≥18 years	n = 124,503	None	↘ Stroke hospitalization (IRR = 0.70, 95% CI: 0.53-0.91)<br>↘ Heart failure hospitalization (IRR = 0.78, 95% CI: 0.65-0.92)<br>↘ PIH (IRR 0.85, 95% CI: 0.74–0.99)<br>↘ ACM (IRR = 0.76, 95% CI: 0.65-0.83)
Wang et al, 2013	Taiwan	Retrospective cohort study	2001-2009	Type 1 & 2	≥65 years	n = 9,025	None	↘ 11% ACH (IRR = 0.78, 95% CI: 0.65-0.92)<br>↘ Intensive care admission (aHR = 0.30, 95% CI: 0.19-0.47)]<br>↘ ACM (aHR = 0.44, 95% CI: 0.36-0.54)

ACH: All-Cause Hospitalization; ACM: All-Cause Mortality; aHR: adjusted Hazard Ratio; CI: Confidence Interval; IH: Influenza Hospitalization; IRR: Incidence Rate Ratio; PIH: Pneumonia and Influenza Hospitalization
