# Supplementary material for: Understanding the gap between guidelines and influenza vaccination coverage in people with diabetes: a scoping review
Source: Front Public Health. 2024 Apr 19;12:1360556. doi: 10.3389/fpubh.2024.1360556 (PMC11066301; doi:10.3389/fpubh.2024.1360556)
Supplement: Supplementary file 2 [file Table_2.PDF]

Table S2. Summary table of studies included on influenza vaccination coverage in people with diabetes per region, country, and influenza season

Reference	Country	Influenza season	Diabetes type	Population age	Population	Estimated coverage (%)
**Americas**						
Monteiro et al, 2018	Brazil	2003<br>2008<br>2015	Type 1 & 2	≥20 years	n = 3,357<br>n = 3,271<br>n = 4,043	47%<br>43%<br>59%
Achtymichuk et al, 2015	Canada	2011-2013	Type 2	≥18 years	n = 2,040	63%
CDC, 2004	US	2003	Type 1 & 2	18-64 years	n = 12,412	49%
Wang et al, 2010	US	2005	Type 1 & 2	≥18 years	n = 1,455	58%
Lu et al, 2016	US	2012-2013	Type 1 & 2	18-64 years	n = 325	58%
O'Halloran et al, 2016	US	2012-2013	Type 1 & 2	18-64 years	n = 22,213	53%
Villarroel et al, 2016	US	2015	Type 1 & 2	≥18 years	NA	62%
**Eastern Mediterranean**						
Alnaheelah et al, 2018	Saudi Arabia	2017-2018	Type 2	NA	n = 353	61%
**Europe**						
Dorner et al, 2011	Austria	1991<br>1999<br>2006-2007	Type 1 & 2	≥15 years	n = 21,959<br>n = 24,612<br>n = 22,999<br>n = 25,563<br>n = 7,005<br>n = 8,469	Men: 6%<br>Women: 6%<br>Men: 13%<br>Women: 14%<br>Men: 22%<br>Women: 21%
De Bruyn et al, 2008	Belgium	2006-2007<br>2007-2008	Type 1 & 2	NA<br>NA	NA<br>NA	46%<br>47%
De Bruyn et al, 2009	Belgium	2008-2009	Type 1 & 2	NA	NA	48%
De Bruyn et al, 2010	Belgium	2009-2010	Type 1 & 2	NA	NA	49%
Clancy et al, 2012	Ireland	2009-2010	Type 1 & 2	48-70 years	n = 198	65%
Tacken et al, 2002	Netherlands	1999	Type 1 & 2	No limits	n = 5,160	85%
Tacken et al, 2015	Netherlands	2008<br>2009<br>2010<br>2011<br>2012<br>2013	Type 1 & 2	>20 years	n ≈ 10,114<br>n ≈ 12,622<br>n ≈ 14,086<br>n ≈ 12,708<br>n ≈ 11,797<br>n ≈ 9,414	85%<br>83%<br>82%<br>79%<br>76%<br>75%
Gorska-Ciebiada et al, 2015	Poland	2012-2013	Type 2	NA	n = 219	27%

Jiménez-García et al, 2005	Spain	1993<br>2001	Type 1 & 2	≥16 years	n = 911<br>n = 1,232	43%<br>49%
Jiménez-García et al, 2008	Spain	2003	Type 1 & 2	≥16 years	n = 1,295	57%
Jiménez-García et al, 2011	Spain	2006	Type 1 & 2	≥16 years	n = 2,156	60%
Rodríguez-Rieiro et al, 2010	Spain	2009	Type 1 & 2	≤59 years	n = 80,440	34%
Jimenez-Trujillo et al, 2015	Spain	2009<br>2011	Type 1 & 2	40-69 years	n = 1,647	44%*
Jimenez-Trujillo et al, 2013	Spain	2010	Type 1 & 2	≥50 years	n = 1,641	65%
Astray-Mochales et al, 2016	Spain	2011-2012<br>2014	Type 1 & 2	≥16 years	n ≈ 3,677<br>n ≈ 3,757	51%<br>52%
Jiménez-García et al, 2017	Spain	2013	Type 2	⩾25 years	n = 2,288	66%
Alvarez et al, 2017	Spain	2016	Type 2	≥18 years	n = 279	40%
Moreno-Fernández et al, 2020	Spain	2017-2018	Type 1	≥18 years	n = 300	55%
Zuercher et al, 2014	Switzerland	2011-2012	Type 1 & 2	≥18 years	n = 514	64%
**Western Pacific**						
Dower et al, 2011	Australia	2008	Type 1 & 2	≥18 years	n = 654	36%
Yang et al, 2017	China	2014-2015	Type 2	≥65 years	n = 158	55%
Shin et al, 2018	Korea	2005-2015	Type 1 & 2	≥40 years	n = 4,540	50%
Yu et al, 2014	Taiwan	2009-2010<br>2010-2011<br>2011-2012	Type 2	≥40 years	n = 691<br>n = 691<br>n = 691	31%<br>33%<br>35%

* Coverage estimated using people from the 2009 and 2011 cohorts
